# Genetic Diversity Impacts Climate‐Induced Species Range Shifts

**DOI:** 10.1111/ele.70345

**Published:** 2026-03-26

**Authors:** Brunno F. Oliveira, Romain Bertrand, Lise Comte, Jonathan Lenoir, Gaël Grenouillet, Lesley T. Lancaster, Jérôme Murienne, Sarah Diamond, Brett R. Scheffers, R. M. W. J. Bandara, Jake A. Lawlor, Nikki A. Moore, Barrett W. Wolfe, Fabricio Villalobos, Sarah R. Weiskopf, Laura M. Thompson, Malin L. Pinsky, Jonathan Rolland

**Affiliations:** ^1^ Centre de Synthèse et D'analyse Sur la Biodiversité – Fondation Pour la Recherche Sur la Biodiversité Montpellier France; ^2^ Departamento de Ecologia Universidade Federal do Rio Grande do Norte Natal Brazil; ^3^ Centre de Recherche sur la Biodiversité et l’Environnement (UMR5300), Centre National de la Recherche Scientifique (CNRS), Institut de Recherche pour le Développement (IRD), Institut National Polytechnique (INP) de Toulouse, Université de Toulouse Toulouse France; ^4^ Conservation Science Partners, Inc Truckee California USA; ^5^ UMR CNRS 7058, Ecologie et Dynamique Des Systèmes Anthropisés (EDYSAN), Université de Picardie Jules Verne Amiens France; ^6^ Institut Universitaire de France Paris France; ^7^ School of Biological Sciences University of Aberdeen Aberdeen UK; ^8^ Department of Biology Case Western Reserve University Cleveland Ohio USA; ^9^ Department of Wildlife Ecology and Conservation University of Florida Gainesville Florida USA; ^10^ Graduate Program in Ecology and Evolution, Rutgers School of Environmental and Biological Sciences Rutgers, The State University of New Jersey New Brunswick New Jersey USA; ^11^ Department of Biology McGill University Montreal Quebec Canada; ^12^ Centre for Marine Socioecology University of Tasmania Hobart Tasmania Australia; ^13^ Institute for Marine and Antarctic Studies University of Tasmania Hobart Tasmania Australia; ^14^ Red de Biología Evolutiva, Instituto de Ecología A.C. Xelapa Mexico; ^15^ National Climate Adaptation Science Center U.S. Geological Survey Reston Virginia USA; ^16^ School of Natural Resources University of Tennessee Knoxville Tennessee USA; ^17^ Department of Ecology & Evolutionary Biology University of California Santa Cruz Santa Cruz California USA

**Keywords:** adaptive capacity, climate change, evolutionary rescue, genetic drift, range contraction

## Abstract

Climate change threatens biodiversity when species cannot tolerate, adapt to, or track shifting environmental conditions to stay within their climatic niches. A major unresolved question is whether and how species' genetic diversity modulates these dynamics, buffering against range contractions or facilitating range expansions. To test this, we integrated the largest global databases of species range shifts and genetic diversity, encompassing 4673 range shift estimates for 1888 species with available genetic data, including insects, arachnids, birds, fish, and plants. We found that range shifting rates were significantly shaped by the interaction of genetic diversity and climate change velocity. Under rapid warming, species with higher genetic diversity exhibited reduced trailing edge contractions, likely reflecting enhanced evolutionary potential or reduced vulnerability to drift. Under moderate warming, species with higher genetic diversity shifted more rapidly at leading edges and range centroids, consistent with greater colonisation ability. Our study provides evidence that genetic diversity potentially enables persistence at the trailing edge and colonisation at the leading edge, with the magnitude of these effects varying depending on the velocity of climate change.

## Graphical abstract

Genetic diversity may buffer species against climate‐driven range contractions and facilitate expansions, but its role at a global scale remains unclear. Using 4673 range shift estimates for 1888 species, we show that higher genetic diversity reduces trailing edge contractions under rapid warming and accelerates leading edge expansions under moderate warming. These results support the prediction that genetic diversity promotes persistence and colonisation, with effects modulated by the velocity of climate change.

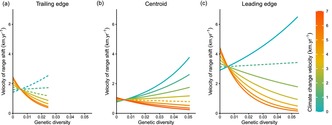

## 1 Introduction

Anthropogenic climate change has impacted biodiversity at all organisational levels, from genes to ecosystems (Pecl et al. 2017; Scheffers et al. 2016). Species can adopt two non‐exclusive eco‐evolutionary strategies in response to climate change. First, they can ‘persist in place’ under new climate conditions through phenotypic plasticity (Dow et al. 2022) or evolutionary adaptation (Bonnet et al. 2022; Thurman et al. 2020). When the velocity of climate warming outpaces these responses, populations decline, geographical ranges contract and species may face extinction (Urban 2015). Second, species can ‘move in space’ to track their climatic niche (Tingley et al. 2009). This often involves geographic range shifts toward higher latitudes and elevations on land, or to deeper habitats in the ocean, allowing populations to remain within suitable environmental conditions (Gruenburg et al. 2025; Lenoir and Svenning 2015; Weinberg 2005). The pace and success of this redistribution depend on both extrinsic (i.e., exposure to environmental conditions and landscape structure) and intrinsic (i.e., species traits influencing eco‐evolutionary responses) factors (Comte et al. 2024). Despite growing evidence of biodiversity redistribution, the role of genetic diversity in modulating climate‐induced range shifts dynamics remains poorly understood (Angert et al. 2020; Diamond 2018; Martin et al. 2023).

Standing levels of genetic diversity within populations can indicate availability of raw genetic material for phenotypic evolution, which in turn underlies a species' ability to respond to environmental change (Barrett and Schluter 2008; O'Brien 1994). By expanding the range of phenotypes available for selection to act upon, higher standing genetic variation enhances the capacity for local adaptation and can ultimately influence the rate and direction of species' range shifts under climate change (Nadeau and Urban 2019). At trailing (warm‐limited) edges, temperature change can surpass species' heat tolerance limits, leading to population declines and range contractions. However, the ability to adapt the thermal niche or to adjust the behavioural thermoregulation can enable persistence, thus slowing range contractions (Frankham 2005; Pérez‐Pereira et al. 2022). This process is well described within the framework of evolutionary rescue, in which adaptation arises from standing genetic variation, *de novo* mutation, or gene flow from other pre‐adapted populations (Bell 2017; Carlson et al. 2014; Frankham 2005; Gomulkiewicz and Holt 1995; Turbek et al. 2023). At leading (cold‐limited) edges (Hampe and Petit 2005), warming can render previously temperature‐limited areas more suitable, making dispersal the primary determinant of range expansion rates. Yet, when sufficient genetic variation is available, evolutionary processes can substantially accelerate range expansion. For instance, spatial sorting can select individuals with highly dispersive traits (Boeye et al. 2013; Miller et al. 2020; Shine et al. 2011) and natural selection can favour fast life‐history traits (Phillips et al. 2010). Greater genetic diversity can further enhance species' potential to cope with novel abiotic and biotic conditions during range expansion, thereby increasing the probability of successful colonisation (Bridle et al. 2014; Lancaster et al. 2015; Lembrechts et al. 2016). Therefore, standing genetic variation may influence the velocity at which species shift their range, slowing trailing‐edge contractions via evolutionary rescue, and enhancing leading‐edge expansions via rapid adaptation to new conditions or faster dispersal.

Eco‐evolutionary responses to climate change are often investigated using genomic approaches developed for model species (Fournier‐Level et al. 2011; Hancock et al. 2011), which typically rely on extensive experiments testing how large‐effect candidate genes influence fitness under novel climatic conditions (Exposito‐Alonso et al. 2019; Yeaman et al. 2016). Other studies use genotype‐environment associations to assess population vulnerability or adaptive potential under future climates (Bay et al. 2018). While these data‐intensive approaches yield detailed insights into the molecular basis of adaptation, they are limited to a few well‐studied taxa. In contrast, the emerging field of macrogenetics explores large‐scale patterns of intraspecific genetic diversity across multiple taxa and regions (De Kort et al. 2021; Leigh et al. 2021; Manel et al. 2020; Miraldo et al. 2016; Yiming et al. 2021). Such studies often quantify genetic diversity as nucleotide diversity or heterozygosity and use these measures as proxies for the standing genetic variation within populations that provides evolutionary potential, including the capacity to respond to environmental change through selection (Barrett and Schluter 2008; Lai et al. 2019; Milot et al. 2020). Genetic diversity estimates may reflect adaptive, preadaptive, and nonadaptive variation shaped by demographic and evolutionary processes (García‐Dorado and Caballero 2021; Teixeira and Huber 2021). However, it remains unclear whether genetic diversity explains why some species rapidly track shifting climates through range expansions or persist locally under changing conditions, while others lag behind or decline.

Recent studies have independently compiled large‐scale databases on species‐level genetic diversity (Fonseca et al. 2023) and range shifts (BioShifts; Comte et al. 2020) for a diversity of taxonomic groups, enabling tests of whether genetic diversity explains species redistribution under climate change (Lenoir et al. 2020). Here, we formulate alternative hypotheses depending on the position within the species range (trailing or leading edges) and the pace of climate change. At the trailing edge, we hypothesise that populations with greater genetic diversity are more likely to persist under warming, thereby reducing the likelihood of trailing‐edge contraction, while populations with lower genetic diversity are more vulnerable and species more prone to trailing edge contraction (Bell 2017; Carlson et al. 2014; Frankham 2005; Pereira et al. 2012). However, warming may proceed faster than the evolutionary responses required to maintain viable populations, potentially limiting trailing edge persistence even if they have relatively high genetic diversity. At the leading edge, whether populations can keep pace with climate change velocity depends on constraints imposed by dispersal capacity, life‐history traits and species interactions (Lawlor et al. 2024). If climate alone limits the range front, higher genetic diversity can accelerate range shifts by enabling evolutionary changes in thermal tolerance (Lancaster et al. 2015). However, when non‐climatic constraints (e.g., biotic interactions, habitat connectivity) predominate, higher genetic variation may facilitate evolution of dispersal, faster life histories, and biotic niche traits required for successful colonisation (Nadeau and Urban 2019). Adaptive genetic variation in traits under selection at leading edges may help expansion keep pace with, or even outpace, the pace of climate change (Lustenhouwer et al. 2017). Nevertheless, rapid warming may outpace the rate of evolutionary responses necessary for species to track shifting conditions.

**FIGURE 1:**
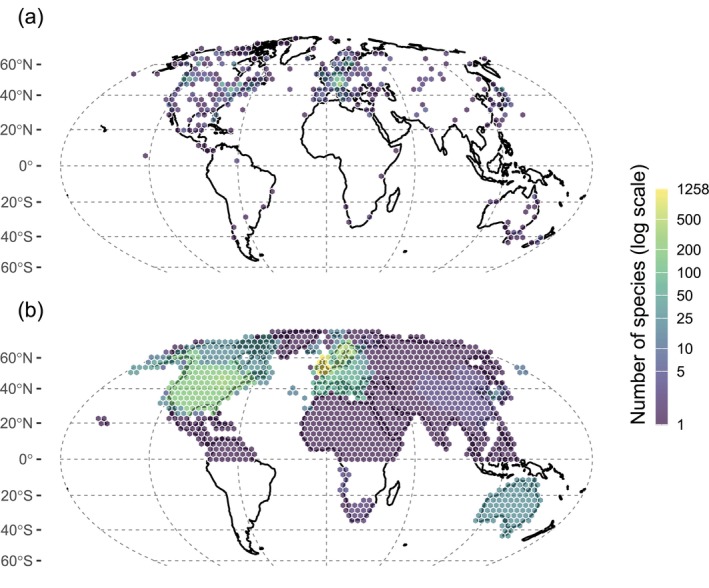
Geographical distribution of genetic and range shift data. (a) Number of species with genetic diversity data within 400‐km equal‐area hexagons. For each species, the location of genetic data was represented by the geographic centroid of all available sampling coordinates (Fonseca et al. 2023). (b) Number of species with range shift velocity data within 400 × 400 km equal‐area hexagons. Range shift data are based on the study area polygons reported in the original sources where range shift estimates were made, as stored in the BioShifts database (Comte et al. 2020). Note that study area polygons can span multiple hexagons depending on their geographic extent, and a single species may be included in multiple study areas (see *Methods*). The two maps differ in their spatial distribution due to the underlying nature of the data: Point‐based sampling centroids for genetic diversity versus spatial polygons for range shift study areas. Hexagons indicate the number of species with available data in each geographic area, shown on a logarithmic scale.

Here, we test whether species genetic diversity is associated with the rate at which they are redistributing their ranges. Specifically, we address three questions: (i) Is genetic diversity associated with range shift velocity? (ii) Does this relationship vary across the positions within the species' range (trailing edge, centroid or leading edge)? and (iii) Does the strength of these associations depend on the degree of exposure to climate change? To answer these questions, we analysed the additive and interactive effects of species genetic diversity, climate change velocity, and species' range position on the velocity at which species have shifted latitudinally over recent decades of warming. We used species‐level nucleotide diversity from a global dataset (Fonseca et al. 2023) as a proxy for genetic diversity, and compared these values to species' range shift rates at up to three distinct range positions (i.e., leading edges, range centroids, or trailing edges), accounting for spatial differences in climate change velocity across these positions (BioShifts, Comte et al. 2020; Figure 1). Our analyses focused on climate‐induced range shift rates, selecting range shifts that occurred in directions consistent with climatic trends, as movements in other directions are less likely to reflect climatic drivers (e.g., land‐use change). We controlled for variation in methodological attributes among studies reporting species range shift rates, as well as for data imbalance, resulting in an analysis covering 4673 range shift observations across 1888 species of insects, arachnids, birds, fishes and plants.

## 2 Material and Methods

### 2.1 Range Shift Data

We gathered marine and terrestrial latitudinal range shift estimates, in km/year, from the BioShifts database (Comte et al. 2020). These estimates were originally collated from published literature sources that used a range of methodological protocols. Previous studies have shown that methodological attributes play an important role in explaining the observed variability in species range shifts (Brown et al. 2016; Dahms and Killen 2023; Lenoir et al. 2020). In addition to range shift estimates, we also extracted information on the position where range shifts were estimated (i.e., at leading edges, for range centroids, or at trailing edges), as well as five methodological attributes describing how the original authors assessed species range shifts, namely: (i) the number of temporal periods used to estimate the velocity of species range shifts (a log‐transformed continuous variable ranging from 2 to 61, mean = 5; LogNTP); (ii) the spatial grain size (an ordinal variable with four levels: small, moderate, large or very‐large; GS); (iii) the data type (a binary variable: abundance‐based vs. occurrence‐based; DTy); (iv) the sampling design (a factor variable with three levels: balanced resampling from opportunistic sampling, raw data, and resurveys from permanent plots; SDes); and (v) the study area extent (a continuous log‐transformed variable; LogSAE). We removed two species, *Agrilus planipennis* and *Chrysodeixis eriosoma* , because their exceptionally high range shift values were computed at the scale of an entire continent. To describe climate change exposure, we also gathered, from the BioShifts database, estimates of the latitudinal velocity of climate change. Climate change velocities were calculated for the estimated range shift period following the methodology from Burrows et al. (2014). Given our focus on climate‐driven range shifts, we excluded cases where species' range movements are opposite to the direction of shifting isotherms, as such mismatches likely reflect other drivers (e.g., land‐use change in terrestrial systems). In the BioShifts database, poleward shifts are encoded as positive velocities and equatorward shifts as negative values, and isotherm shifts follow the same sign convention (negative for equatorward, *n* = 136; positive for poleward, *n* = 4673). We therefore restricted our analysis to cases in which species shifted in the same direction as isotherms. For example, observations of species moving equatorward while temperatures shifted poleward were excluded because they are unlikely to represent responses to climate warming, which is the focus of this study.

### 2.2 Genetic Data

We extracted genetic diversity data from Fonseca et al. (2023), calculated as a species‐average value of intraspecific nucleotide diversity. In that study, nucleotide diversity was calculated from aggregated georeferenced mitochondrial and chloroplast DNA for animals and plants, respectively, using *phylogatR* (Pelletier et al. 2022; https://phylogatr.osc.edu). The *phylogatR* platform retrieves genetic and geographic data from the GBIF (https://www.gbif.org/), GenBank (https://www.ncbi.nlm.nih.gov/genbank/) and BOLD (http://www.boldsystems.org/) databases. After removing species with fewer than five sequences contributing to a mean estimate, 38,134 species remained. The vast majority (> 98%) of the genes recorded in their database were from the cytochrome c oxidase subunit I (COI) for animals and ribulose‐1,5‐bisphosphate carboxylase/oxygenase large subunit (RBCL) for plants. The authors report no major differences in average genetic diversity among marker types. For each species, the centroid of all sampling localities, based on the geographical coordinates of the genetic data extracted from *phylogatR*, was used to represent its spatial distribution.

### 2.3 Pairing Range Shift Data With Genetic Data

To match the range shift and genetic diversity databases, we harmonised species names using the R packages *rgbif* and *bdc*. Taxonomic classes with less than 10 species were removed to avoid taxonomic imbalance. After matching taxonomies, our final dataset included 4673 range shift estimates covering 1888 species with genetic data from 7 major taxonomy classes: 1751 range shift estimates for 1002 species of insects (Insecta); 1611 range shift estimates for 342 species of birds (Aves); 830 range shift estimates for 256 species of plants belonging to three different classes (Magnoliopsida, Polypodiopsida, and Liliopsida); 248 range shift estimates for 154 species of arachnids (Arachnida); and 235 range shift estimates for 134 species of fishes (Actinopterygii). Range shift and genetic diversity data were merged at the species level, regardless of the geographic location of the genetic sampling. Because coordinates for individual observations contributing to the averaged genetic diversity were not available, we could not determine whether the genetic data were representative of specific positions within each species' range.

### 2.4 Modelling Framework

In order to test the association between genetic diversity and the velocity of species latitudinal range shifts, we used generalised linear mixed‐effects models (GLMMs). We fitted the absolute value of the rate at which species are shifting along the latitudinal gradient (|LRS|; the response variable in km/year) against a set of fixed‐effects variables including species genetic diversity (GD), the absolute value of the velocity at which isotherms are shifting along the latitudinal gradient (|VIS|), and the position within the species range (POS; a factor variable with three levels for leading edges, trailing edges, and centroids of ranges, with the latter as the reference level). We included all two‐way and the three‐way interaction terms among GD, |VIS|, and POS to evaluate whether the effect of genetic diversity on range shift velocity depends on exposure to climate change (|VIS|) and on the specific part of the range where shifts are observed (POS).

The methodological approaches used to quantify and analyse species' range shifts in the original studies registered in the BioShifts database, such as survey techniques, time intervals, and spatial resolution, strongly affect the variability of observed range shifts across studies (Lenoir et al. 2020). As a consequence, we included five methodological variables (LogNTP, LogSAE, GS, DTy and SDes; see “Range shift data”) as fixed effect variables in our model formula to capture this variability.

In addition, our dataset comprises multiple species within each taxonomic class, with similar life histories, eco‐evolutionary characteristics, and sensitivity to climate change. To control for this non‐independence among related species, we included the taxonomic class (Class) as a random‐intercept term in our model formula.

Therefore, our full model formula was:

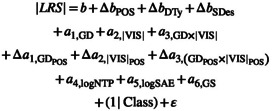




Here, *b* is the intercept, representing the combination of reference levels of the three categorical variables listed in the fixed effects. Reference levels were set to “range centroid”, “abundance sampling design” and “occurrence data type” for POS, SDes and DTy, respectively. The terms *b*POS, *b*DTy, and *b*SDes represent the deviations, from the reference level, of the other levels of these three categorical variables. The coefficients *a*₁ to *a*₆ correspond to the effects of the continuous predictors. Specifically, *a*₁ to *a*₃ represent the effects of GD, |VIS|, and their interaction at the range centroid. Δ*a*_1,POS_, Δ*a*_2,POS_, Δ*a*_3,POS_ are interaction terms that capture deviations in these effects at the leading and trailing edges relative to the range centroid. The term (1∣Class) denotes a random intercept and ε the residual error. Note that genetic diversity was extracted from the global macrogenetic database of Fonseca et al. (2023) as species‐level mean nucleotide diversity, representing an aggregate measure across all available populations within each species. The GD variable was therefore treated as a species‐level covariate in the model, consistent with the study's cross‐species comparative scope. We computed the marginal effects of genetic diversity, interacting with climate change velocity, on the absolute velocity of latitudinal species range shifts at the other two range positions as *a*_1_ + *a*_1,POS_ + (*a*_3_ + *a*_3,POS_)|VIS|.

Because we modelled absolute range shift rates (positive values only), we fitted our GLMM formula using a Gamma distribution of errors with a log link function. The |VIS| and GD variables were standardised to *z*‐scores to allow direct comparisons of model coefficients. In addition, the number of range shift observations per position was imbalanced (2298, 2082, and 295 observations for the center of the range, leading edge and trailing edge, respectively). To account for this imbalance, we weighted observations in the GLMM formula as follows:

$$W_{i,\mathrm{POS}}=\frac{1}{N_{\mathrm{obs}\mathrm{sp},\mathrm{POS}}}\times \frac{1}{N_{\mathrm{sp},\mathrm{POS}}}\times \frac{1}{3}$$

with *i* being the *i*th observation (*n* = 4673), *N*_obs*sp*,POS_ being the number of range shift observations found in the dataset for the focal species at a focal range position, *N*_*sp*,POS_ being the total number of species at a given range position, and ⅓ representing the equal contribution of each of the three range positions.

We tested for collinearity among predictor variables using the variance inflation factor. This issue is unlikely to have impacted the model substantially because variance inflation factors were never greater than 3.

Because the usefulness of *p*‐values for hypothesis testing in GLMMs is still debated in the scientific literature (Bates et al. 2015), we used a bootstrap modelling approach (*N* = 12,000 bootstrap iterations) to compute confidence intervals as an alternative. First, for each bootstrap iteration, we randomly selected n observations (*n* = 4673) with replacement. Second, we computed a weight for each observation as presented above. Third, we fitted the GLMM with maximum likelihood parameter estimates. Finally, because some of the 12,000 bootstrap iterations did not converge due to singularity issues, we randomly selected 10,000 converging bootstrapped models. We calculated both the mean effect of each fixed variable (including the marginal effects of genetic diversity across values of POS and |VIS|), as well as their respective 95% confidence intervals, from this set of bootstrapped models. The significance of each variable was determined from the bootstrap distribution for the two alternative hypotheses of its mean coefficient estimate being greater or lower than zero (i.e., the null hypothesis). To assess the goodness‐of‐fit of the models, we also computed the mean marginal (i.e., variance explained by the fixed effects) *R* ^2^ values using the lognormal method, which is recommended for models with the logarithmic link function, as well as their respective 95% confidence intervals (CIs) from the set of bootstrap iterations. Using a hierarchical variation partitioning approach, we quantified the relative contribution of each predictor, interaction terms and combination of method variables, toward the total marginal *R* ^2^. This last analysis was not performed with bootstrap due to computation time limitation.

We fitted additional models to test if our results were sensitive to the imbalance in the number of observations per range position. Specifically, we subset the full dataset for each range position and fitted three independent GLMMs, one for each range position (trailing edge, centroid, and leading edge). We repeated the same statistical approach as described above but removed the three‐way interaction term with POS (*a*_3_(GD_POS_ × |VIS|_POS_)) as well as the two sets of two‐way (*a*_1_(GD_POS_) + *a*_2_(|VIS|_POS_)) interaction terms involving POS.

**FIGURE 2:**
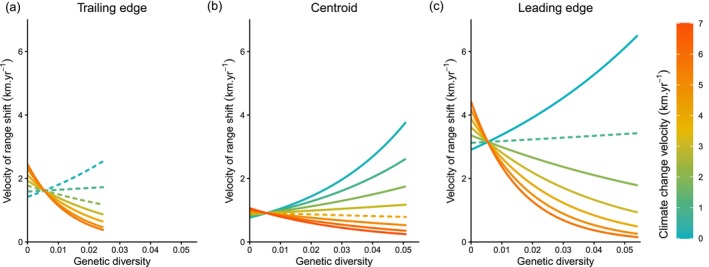
Marginal effects of the interaction between climate change velocity and genetic diversity on species range shifts. Predicted effects of genetic diversity on species range shifts across different magnitudes of climate change velocity at: (a) trailing edges, (b) range centroids, and (c) leading edges. Solid and dotted lines indicate significant and non‐significant relationships, respectively (see *Methods*). See also Figures S1 and S2 for corresponding raw data plotted on the same axes.

Predictions shown on Figure 2 are representative of species range shifts inferred from a representative combination of methodological attributes set to occurrence‐based observations, a balanced sampling, a moderate spatial resolution, a spatial extent of the study area of 1238 km^2^ and a number or temporal period of sampling of 4.9 periods (i.e., average values found in the BioShifts database).

We performed additional sensitivity analyses to assess the robustness of our results to the methodological choice of using a single GLMM with range position as a fixed‐effect factor. To this end, we fitted three independent GLMMs, each applied separately to a different range position, and found consistent results across approaches. We also evaluated whether historical legacies (using latitude as a proxy) could account for residual variability in range shifts not explained by genetic diversity and climate change velocity. First, we tested whether latitude explained the residuals of a baseline model that included climate change velocity, genetic diversity, range position, methodological differences (Methods), and a random effect of taxonomic class (Class)—that is, whether latitude captured variance not accounted for by these predictors. Then, we reversed the approach: we fitted a model including latitude and Methods (with Class as a random effect), and tested whether climate change velocity and genetic diversity explained the residuals of this model—that is, the variance not explained by latitude.

Although other ecological and life‐history factors not included in our models may influence range shift rates and contribute to the unexplained variation (Comte et al. 2024; Lawlor et al. 2024), our analysis focused on species‐level genetic diversity, velocity of climate change, and range position while accounting for imbalances among studies and taxa through weighting and random effects. The use of bootstrapped confidence intervals and standardised coefficients provides a transparent estimate of the explanatory power of the predictors included in our models.

All statistical analyses were performed in R version 4.4.2 (R Core Team 2024) using the *lme4* (Bates et al. 2015), *glmmTMB* (Brooks et al. 2017), *MuMIn* (Bartoń 2017) and *glmm.hp* (Lai et al. 2022) packages. The R‐code used to run all analyses in this study is available at https://github.com/bioshifts/genetic_diversity_range_shifts.

## 3 Results

Our models explained, on average, 50% of the observed variance in the absolute value of species' range shift velocity (*R* ^2^_marginal_: 95% CI = 47%, 53%). The methodological approaches used to quantify and analyse species' range shifts contributed to the largest proportion of the total explained variance (56%), followed by range‐position effects and its interactions (38.4%; Table S1). Additionally, genetic diversity and its interactions accounted for ~10% of the variance and climate change velocity and its interactions explained 9.3% of the observed variance (Table S1). Our results also suggest that genetic diversity significantly impacts the absolute velocity of species' range shifts, primarily through interactions with climate change velocity and range position (trailing edge, centroid, or leading edge), which account for 83.5% of the total contribution of genetic diversity (Table S1). Specifically, at the range centroid (i.e., our reference range position), we found a negative interaction between genetic diversity and climate velocity (Estimate = −0.086; CI = −0.122, −0.047), showing that species with higher genetic diversity tended to shift more slowly under fast climate change velocities, but more quickly under slow climate change velocities (Figure 2; Table S2).

The effect we reported at the range centroid was enhanced at the trailing edge, where genetic diversity had a consistently negative and significant effect on range shift velocity under fast climate change velocities (i.e., 2.7–5.6 km/year; Figure 2a; Table S3). This finding supports the hypothesis that high genetic diversity promotes species persistence by buffering against range contractions. For instance, at high climate change velocities, the expected rate of species range shift declined from an average of 1.99 km/year (range: 1.73–2.45 km/year) for species with low genetic diversity (i.e., lower than 0.003) to 0.8 km/year (range: 0.38–1.24 km/year) for species with higher genetic diversity (i.e., higher than 0.015) (Figure 2a).

At the leading edge, the effect of genetic diversity on the absolute value of range shift velocity was positive relative to the centroid baseline, indicating that species with higher genetic diversity tend to shift faster at the leading edge compared to the centroid when climate change velocity was slow (0–0.7 km/year at the leading edge and 0.1–3 km/year at the range centroid; Figure 2b,c; Table S3). For instance, under climate change velocities below 1 km/year, the expected rate of range expansion at leading edges increased from 3.02 km/year (prediction interval: 2.99–3.1 km/year) for species with low genetic diversity (i.e., lower than 0.003) to over 4.39 km/year (prediction interval: 3.37–6.51 km/year) for species with high genetic diversity (i.e., higher than 0.015). This pattern supports the theoretical prediction that high genetic diversity can boost range expansions. However, under fast climate change velocities (above 2 km/year at the leading edge and above 4.4 km/year at the centroid), higher genetic diversity was associated with slower range shifts at these range positions (Figure 2b,c; Table S3).

We additionally tested whether historical legacies (specifically captured by latitude) could account for some of the variability in range shift velocities that remained unexplained by genetic diversity and climate change velocity. Our results showed that latitude did not significantly improve the explanatory power beyond that provided by genetic diversity and climate change velocity (Tables S4 and S5). Finally, our results and conclusions remained consistent when fitting three independent GLMMs, each applied separately to a different range position, suggesting that our findings are not sensitive to this modelling choice (Figure S3).

## 4 Discussion

Species are shifting their ranges unevenly under climate change, yet the mechanisms underpinning this variation remain unclear. While previous studies have mapped spatial gradients and environmental correlates of genetic diversity (De Kort et al. 2021; French et al. 2023; Theodoridis et al. 2020), the eco‐evolutionary implications of genetic diversity for species redistribution under climate change remain largely unresolved. In conservation genetics, the role of genetic variation in promoting both local persistence under climate stress and successful colonisation beyond current range limits has long been debated (Lancaster 2022; Nadeau and Urban 2019; Waldvogel et al. 2020), yet robust empirical tests across taxa and continents have been missing. By synthesising two of the largest global datasets on genetic diversity and documented species' range shifts, our study provides the first broad‐scale empirical evidence that species' genetic diversity is a key predictor of the velocity of their climate‐driven range shifts across multiple taxonomic groups.

Our results show that genetic diversity modulates species' range shifts through its interactions with climate change velocity and range position, accounting for about 10% of the total variance explained by our models. Notably, interaction terms account for 83.5% of the variance attributed to genetic diversity, indicating that its influence arises not in isolation, but through modulating species' responses to climatic exposure, with effects varying across species' range positions. These findings are consistent with trait–environment frameworks in which range shifts emerge from the interplay between climatic exposure and species' intrinsic traits (Comte et al. 2024). Methodological variability was the dominant source of explained variation, in line with a previous study using the same global range shift dataset (Lenoir et al. 2020). Although other ecological and life‐history traits not included in our models may influence range shift rates and contribute to the unexplained variation (Comte et al. 2024; Lawlor et al. 2024), our results nonetheless demonstrate that genetic diversity does explain a substantial part of the variance in species' climate‐driven range shifts at broad scales.

We found that genetic diversity potentially enhanced species persistence under climate change by mitigating contractions at the trailing edge of species ranges. According to evolutionary rescue theory, populations primarily adapt to novel environments through pre‐existing alleles (i.e., standing genetic variation), which provides an immediate pool of beneficial options for rapid adaptation (Bell 2017; Carlson et al. 2014; Gomulkiewicz and Holt 1995), rather than relying on *de novo* mutations. Moderate levels of gene flow from core to edge populations can further boost evolutionary potential by introducing genetic variation that can favour faster adaptation to environmental change (Duputié et al. 2012; Rolland et al. 2015). These insights have important implications for conservation, as high genetic diversity is considered key to enhancing population resilience and reducing extinction risk (Donelson et al. 2019; Shaw et al. 2025), particularly at warm edges (Nicastro et al. 2013). Genetic diversity is generally lower in threatened species (Petit‐Marty et al. 2021), and because population decline is a primary criterion for assessing threat status (Mace et al. 2008), such declines may also signal vulnerability from reduced genetic variation (Hoban et al. 2020). For instance, low genetic diversity and rapid population declines have been found in Tasmanian devils ( *Sarcophilus harrisii* ) (Lachish et al. 2011) and koalas (*Phascolarctos cinereus*; Johnson et al. 2018), where reduced diversity increases vulnerability to diseases. In contrast, in corals (e.g., *Acropora hyacinthus* ), genetic diversity has been shown to facilitate adaptive shifts in thermal tolerance through selection on heat‐resistant genotypes in the warmest reef locations, potentially delaying climate‐driven range contractions (Bay and Palumbi 2014). In our dataset, passerine species such as golden‐crowned kinglet ( *Regulus satrapa* ) and hermit thrush ( *Catharus guttatus* ), as well as bumblebees ( *Bombus cryptarum* , *B. hypnorum* and *B. monticola* ), exhibited relatively slower trailing edge contractions (defined as being below the lower quantile of trailing edge velocity), alongside high genetic diversity (defined as being above the upper quantile of genetic diversity). These species often display wide habitat ranges (Graham et al. 2020; Maebe et al. 2019; Ruegg and Smith 2002), and evolution of traits conferring habitat flexibility may further slow fitness declines at warm edges, resulting in an extinction debt (Nadeau and Urban 2019). In contrast, in our dataset, shorebirds such as *Lymnocryptes minimus* and *Tringa erythropus* exhibited lower genetic diversity alongside much faster trailing edge contractions, supporting previous evidence that shorebird are highly sensitive to habitat loss and display severe climate‐driven population declines (Smith et al. 2023). Maintaining multiple populations and facilitating gene flow among them may simultaneously boost genetic diversity and enhance the probability of genetic rescue, while preserving locally adapted alleles that are unique to edge environments (Hargreaves and Eckert 2019; Sexton et al. 2011).

Unlike the patterns observed at the trailing edge, where higher genetic diversity was consistently associated with slower range contractions, our findings revealed a more complex relationship at the range centroid and at the leading edge. Specifically, the effect of genetic diversity on species' ability to track climate change was strongly contingent on the rate of climate change exposure. This non‐linear relationship revealed a critical threshold: while genetic diversity was positively associated with faster centroid shifts and range expansions under low to moderate climate change velocities, this association reversed when climate change was fast (above 2 km/year at the leading edge and above 4.4 km/year at the centroid). Therefore, under rapid climate change, it was the less genetically diverse species that shifted faster.

Range expansion models suggest that natural selection favours faster dispersal that can accelerate niche tracking at the leading edge (Atkins and Travis 2010; Boeye et al. 2013; Miller et al. 2020). Additionally, reduced intra‐ and interspecific competition can relax constraints on trait evolution, enabling selection to favour fast life‐history traits, such as short generation times and high fecundity, at the leading edge (Phillips et al. 2010). In general, species with fast life‐history traits are expected to maintain higher genetic diversity (Romiguier et al. 2014). In our dataset several species that exhibited rapid leading edge expansions were Lepidoptera (upper quantile of leading edge shifts; *N* = 208 out of 439) from the families Noctuidae (e.g., *Autographa jota*, *Cosmia trapezina*, *Ipimorpha subtusa*) and Nymphalidae (e.g., *Araschnia levana*, *Argynnis paphia*, *Nymphalis xanthomelas*), which typically exhibit fast life‐history strategies and high dispersal capacity. However, these rapidly expanding species also showed lower genetic diversity compared to other Lepidoptera in our dataset (GLM: Estimate = −64.68; CI = −112.66, −16.70). Although our genetic diversity estimates are not necessarily obtained from the location where the expansion is occurring, these species' life histories may expose them to drift and bottlenecks throughout their ranges (Garnier and Lewis 2016; Gilbert et al. 2018). For example, serial founder effects and strong genetic drift may have reduced genetic diversity at the leading edge relative to range centroid populations in invasive honey bees (e.g., *Apis cerana* ; Hagan et al. 2024), and slowed down range expansions in damselflies (e.g., *Coenagrion scitulum*; Swaegers et al. 2013) and herbs (e.g., *Argentina anserina* ; Cisternas‐Fuentes and Koski 2023). The extent to which dispersal and life‐history traits alone are sufficient to ensure successful range shifts remains debated (Beissinger and Riddell 2021; Estrada et al. 2016). A better understanding of their capacity to sustain long‐term population viability and evolutionary potential under climate change will require more detailed trait data and population genomics across taxa (Comte et al. 2024).

The estimates of genetic diversity used here (Fonseca et al. 2023) are based on mitochondrial or chloroplast markers which are representative of both non‐adaptive and adaptive standing genetic variation, rather than a direct measure of adaptive variation. While a small fraction of substitutions may be under positive selection (Bazin et al. 2006; Dowling et al. 2008; James et al. 2016), these markers rather primarily reflect neutral processes, geographical structure and demographic history, which may themselves affect the amount of standing genetic variation, and ultimately indirectly impact the ability to adapt to changing conditions (Barrett and Schluter 2008; Lai et al. 2019). As in most macrogenetic studies, organellar markers offer the opportunity of a global, multi‐taxa comparison of intraspecific diversity (e.g., Fonseca et al. 2023; Manel et al. 2020; Theodoridis et al. 2020), but the genes under selection are likely different among clades. More research is also needed to clarify the conditions under which organellar diversity is correlated to—adaptive and non‐adaptive—nucleotide diversity at the genome scale (Clark and Pinsky 2024; Leigh et al. 2021; Schmidt and Garroway 2021; Toews and Brelsford 2012).

Our results indicate a positive association between species' genetic diversity and range‐shift dynamics, but they do not establish a causal link because multiple ecological, evolutionary, and demographic processes can shape both genetic variation and the pace of range shifts.

First, historical legacies, such as past climatic fluctuations, long‐term population dynamics, and historical dispersal constraints, can shape both genetic diversity and traits. Some high‐latitude species inherently exhibit low genetic variation due to past climatic oscillations and post‐glacial range dynamics, which involved repeated range expansions and bottlenecks (Dynesius and Jansson 2000; Fonseca et al. 2023; French et al. 2023; Hewitt 2000; Manel et al. 2020; Rolland et al. 2020). These species often possess traits that promote range expansion, as historical climate dynamics may have acted as a selective filter favouring high dispersal ability, rapid population growth, and ecological generalism (Chuang and Peterson 2016; Comte et al. 2024; Phillips et al. 2010; Shine et al. 2011). To assess whether such historical legacies could explain variation in observed range shifts, we tested latitude as a proxy for historical effects, finding it did not significantly improve model performance beyond genetic diversity and climate change velocity (Tables S3 and S4). This suggests that the negative association between range shift velocity and genetic diversity under rapid climate change is unlikely to be driven solely by historical legacies.

Second, recent demographic processes also influence genetic diversity. Environmental and anthropogenic pressures can drive range contractions and expansions that reduce genetic variation through demographic bottlenecks (Shaw et al. 2025; Turnock et al. 2025). Larger populations should harbour higher genetic diversity and decline more slowly (Petit‐Marty et al. 2021; but see Bazin et al. 2006), which could generate a negative correlation between genetic diversity and trailing‐edge contraction even in the absence of in situ adaptation. However, our study characterises broad‐scale relationships between genetic diversity and range shift dynamics at the species‐level, rather than population‐level. Genetic diversity data were aggregated across multiple populations, but not necessarily measured where population‐level range shifts were estimated. An appropriate design, combining genome sequencing and demographic estimates from the same populations at range edges, would be required to disentangle the relative contributions of standing genetic variation, adaptive responses, and demographic correlates to trailing‐edge persistence.

Our analysis leverages recent advances in macrogenetics and biodiversity redistribution to provide wide‐scale evidence that genetic diversity plays a key role in mediating species' range shift dynamics in response to climate change. We show that genetic diversity is positively correlated with species' ability to track shifting climates, particularly at intermediate levels of climate change velocity, while also to persist at trailing edges under rapid climate change. These findings underscore the value of genetic diversity not only as a long‐term evolutionary reservoir but also as a short‐term buffer against environmental change. However, species redistribution is a complex process influenced not only by evolution but also by behavioural plasticity, phenological shifts, physiological acclimation, and species interactions (Comte et al. 2024; Pinsky et al. 2022; Seaborn et al. 2021; Thurman et al. 2022). Disentangling the relative contributions of these mechanisms remains a major challenge in global change biology. Moving forward, there is a need for integrative approaches that combine population genetic data with demographic, ecological, and environmental information across spatial and temporal scales. Such integration is needed for predicting species responses to accelerating environmental change and for informing conservation strategies aimed at safeguarding biodiversity resilience in the Anthropocene.

## Author Contributions

The study was conceived and designed by all authors, with significant contributions from B.F.O., R.B., L.C., J.L., L.T.L., J.M., S.D., M.L.P., and J.R. Methodological design was developed primarily by B.F.O., R.B., L.C., J.L., G.G., J.M., M.L.P., and J.R. All analyses were carried out, and figures were designed, by B.F.O. and R.B., with regular and substantial input from J.R. and M.L.P. The original draft of the manuscript was written by B.F.O., J.R. and R.B, then revised by B.F.O. and J.R., with significant contributions from R.B., L.C., J.L., G.G., J.M., and M.L.P. All authors contributed to reviewing and editing the manuscript and approved the final submitted version.

## Funding

This work was supported by Agence Nationale de la Recherche (Grants CEBA: ANR‐10‐LABX‐25‐01, CPJ [R.B.]: ANR‐22‐CPJ2‐0037‐01, JCJC [J.R.]: ANR‐23‐CE02‐0005‐01, and TULIP: ANR‐10‐LABX‐0041), French Foundation for Research on Biodiversity, National Science Foundation (Grants DEB‐2129351, DEB‐2343787, and OISE‐1743711), and the Centre for the Synthesis and Analysis of Biodiversity (CESAB).

## Conflicts of Interest

The authors declare no conflicts of interest.

## Supporting information


**Figure S1:** Predictions of species range shifts across genetic diversity and climate change velocities. Predictions at the trailing edge (a), centroid (b) and leading edge (c) are computed from the GLMM model presented in Figure 2 in the main text. The colour of the envelope represents the density of observations.
**Figure S2:** Marginal effects of the interaction between climate change velocity and genetic diversity on species range shifts. Predicted effects of genetic diversity on species range shifts across different magnitudes of climate change velocity at: (a) trailing edges, (b) range centroids, and (c) leading edges. Solid and dotted lines indicate significant and non‐significant relationships, respectively (see *Methods*). This figure corresponds to Figure 1 in the main manuscript, with raw data plotted on the same axes.
**Figure S3:** Predicted effects of genetic diversity on species range shifts across different magnitudes of climate change velocity (a) at trailing edges, (b) range centroid and (c) leading edges. Predictions have been computed from separate GLMMs for each range position. Solid and dotted lines show respectively significant and non‐significant relationships (see *Methods* for further details on the modelling framework).
**Table S1:** Hierarchical partitioning of explained variance in species' range shift velocity. The table shows the relative contribution of each predictor, interactions, and methodological variables to the total marginal *R* ^2^ of the generalised linear mixed‐effects models. Rows indicate predictor variables: |VIS|, absolute velocity of isotherm shift for centroid shifts; GD, Genetic diversity; POS, location within the species range; Methods, the combined effect of all methodological variables included in the model (LogNTP, LogSAE, GS, DTy, SDes). Interactions are indicated by colon (“:”) between variable names. Columns indicate: Unique, the independent contribution of each term; Average share, the shared average contribution; Individual, combined contribution of unique and shared variance; Ind. perc. (%), percentage of total explained variance. The total variance explained by a single variable includes its unique contribution plus all shared contributions through interactions. For example, the variance explained by GD is calculated as 1.64 + 4.33 + 1.70 + 2.28 = 9.95% (with interaction explaining: 4.33 + 1.70 + 2.28 = 8.31%). The variance explained by |VIS| and POS are equal to 9.27% (interaction: 6.77%) and 38.04% (interaction: 9.5%), respectively.
**Table S2:** Summary of the effect estimates computed from the 10,000 bootstrapped models. The model explains 50% of the species range shifts on average (95% CI from 47% to 53%). The * indicates significant values (*p* < 0.05). Right‐aligned variables indicate deviations from the left‐aligned effect displayed above. Although multiple measurements of range shift may be available for the same species at different study areas, genetic diversity was treated as a species level covariate. Refer to the methods session for further details on the modelling approach and included covariates.
**Table S3:** Ranges of climate change velocities for which genetic diversity has a significant negative or positive effect on the velocity of species range shifts.
**Table S4:** Summary of models testing whether latitude covaries with the relationship between range shift velocity, climate change velocity, genetic diversity, and range position. The baseline model includes all two‐ and three‐way interactions among climate change velocity, genetic diversity, and range position. Additional models (Latitude 1 and Latitude 2) evaluate the role of latitude either as an additive covariate or through a full four‐way interaction. Model performance is compared using *R* ^2^ and AIC values, with lower AIC indicating better fit and higher *R* ^2^ indicating greater explanatory power.
**Table S5:** Summary of models testing whether latitude, climate change velocity, and genetic diversity independently explain residual variation in species' range shift velocity. We first tested whether latitude explains the residuals of a baseline model that included climate change velocity, genetic diversity, range position, methodological variables (Methods), and a random effect of taxonomic class (Class) (i.e., whether latitude accounts for variance not explained by these predictors). Next, we fitted a model including latitude and methodological variables, and a random effect of taxonomic class (Class), then tested whether climate change velocity and genetic diversity explain the residuals of this model (i.e., the variance unexplained by latitude). *R* ^2^ values are reported for all models to indicate the proportion of variance explained. Overall, climate change velocity and genetic diversity explained more of the variation not captured by latitude than latitude explained of the variation not captured by climate change velocity and genetic diversity.
**Dataset: S1** Summary of the number of species and range shifts per taxonomic class, order and family.

## Data Availability

All data used in this study is publicly available. Genetic data from Fonseca et al. (2023) is available from https://github.com/emanuelmfonseca/global_genetic_diversity, and range shift data from the BioShifts database (Comte et al. 2020) is accessible from https://doi.org/10.6084/m9.figshare.7413365.v1. R‐code to reproduce all analyses and figures in this study is available from https://github.com/bioshifts/genetic_diversity_range_shifts.
