# Supplementary material for: Genetic Diversity Impacts Climate‐Induced Species Range Shifts
Source: Ecol Lett. 2026 Mar 26;29(4):e70345. doi: 10.1111/ele.70345 (PMC13022066; doi:10.1111/ele.70345)
Supplement: Supplementary file 1 — Figure S1: Predictions of species range shifts across genetic diversity and climate change velocities. Predictions at the trailing edge (a), centroid (b) and leading edge (c) are computed from the GLMM model presented in Figure 2 in the main text. The colour of the envelope represents the density of observations. Figure S2: Marginal effects of the interaction between climate change velocity and genetic diversity on species range shifts. Predicted effects of genetic diversity on species range shifts across different magnitudes of climate change velocity at: (a) trailing edges, (b) range centroids, and (c) leading edges. Solid and dotted lines indicate significant and non‐significant relationships, respectively (see Methods). This figure corresponds to Figure 1 in the main manuscript, with raw data plotted on the same axes. Figure S3: Predicted effects of genetic diversity on species range shifts across different magnitudes of climate change velocity (a) at trailing edges, (b) range centroid and (c) leading edges. Predictions have been computed from separate GLMMs for each range position. Solid and dotted lines show respectively significant and non‐significant relationships (see Methods for further details on the modelling framework). Table S1: Hierarchical partitioning of explained variance in species' range shift velocity. The table shows the relative contribution of each predictor, interactions, and methodological variables to the total marginal R 2 of the generalised linear mixed‐effects models. Rows indicate predictor variables: |VIS|, absolute velocity of isotherm shift for centroid shifts; GD, Genetic diversity; POS, location within the species range; Methods, the combined effect of all methodological variables included in the model (LogNTP, LogSAE, GS, DTy, SDes). Interactions are indicated by colon (“:”) between variable names. Columns indicate: Unique, the independent contribution of each term; Average share, the shared average contribution; Individual, comb [file ELE-29-0-s001.pdf]

Supplementary Material for

Genetic diversity impacts climate-induced species range shifts

Brunno F Oliveira, Romain Bertrand, Lise Comte, Jonathan Lenoir, Gael Grenouillet, Lesley T Lancaster, Jerome Murienne, Sarah Diamond, Brett R Scheffers, RMWJ Bandara, Jake A Lawlor, Nikki A Moore, Barrett W Wolfe, Fabricio Villalobos, Sarah R Weiskopf, Laura M Thompson, Malin L Pinsky, Jonathan Rolland

Corresponding author: Jonathan Rolland
Email: jonathan.rolland@utoulouse.fr

This PDF file includes:

Figures S1 to S3

Tables S1 to S5

Dataset S1

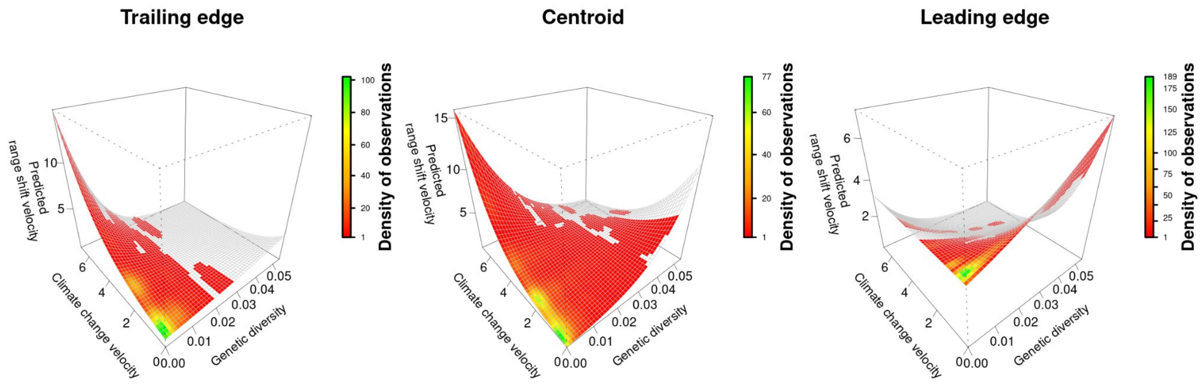

Figure S1. Predictions of species range shifts across genetic diversity and climate change velocities. Predictions at the trailing edge (a), centroid (b) and leading edge (c) are computed from the GLMM model presented in Figure 2 in the main text. The colour of the envelope represents the density of observations.

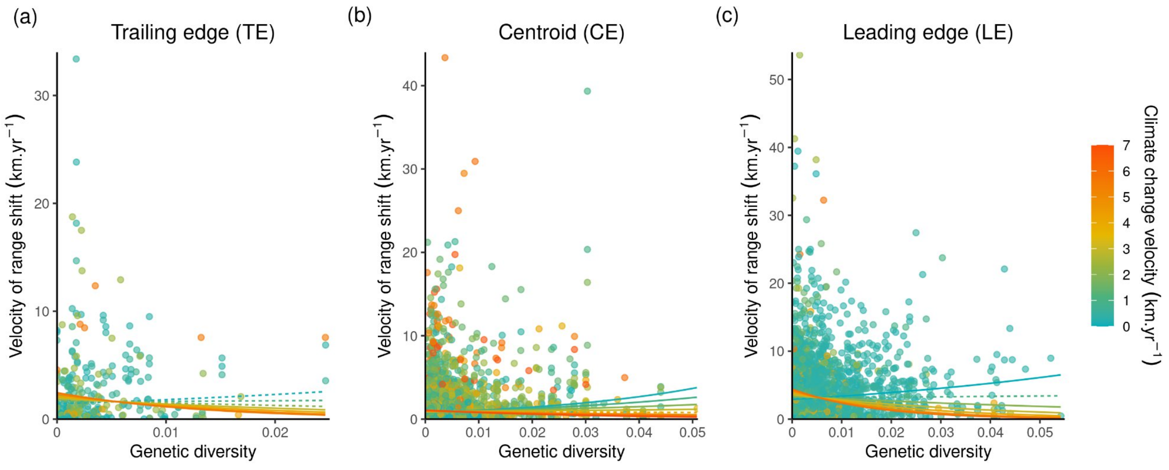

Figure S2. Marginal effects of the interaction between climate change velocity and genetic diversity on species range shifts. Predicted effects of genetic diversity on species range shifts across different magnitudes of climate change velocity at: (a) trailing edges, (b) range centroids, and (c) leading edges. Solid and dotted lines indicate significant and non-significant relationships, respectively (see *Methods*). This figure corresponds to Figure 1 in the main manuscript, with raw data plotted on the same axes.

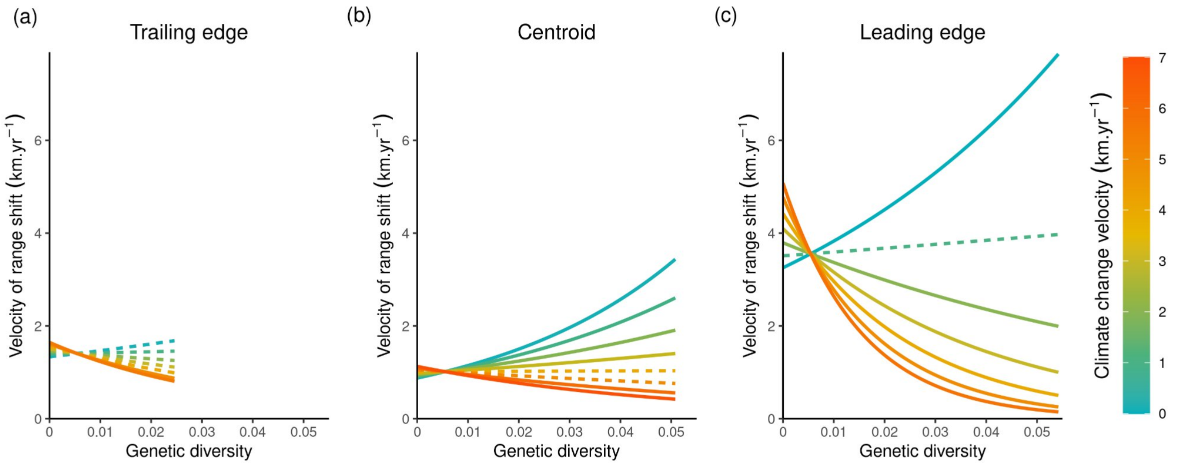

Figure S3. Predicted effects of genetic diversity on species range shifts across different magnitudes of climate change velocity (a) at trailing edges, (b) range centroid and (c) leading edges. Predictions have been computed from separate GLMMs for each range position. Solid and dotted lines show respectively significant and non-significant relationships (see *Methods* for further details on the modeling framework).

Table S1. Hierarchical partitioning of explained variance in species' range shift velocity. The table shows the relative contribution of each predictor, interactions, and methodological variables to the total marginal R^2 of the generalized linear mixed-effects models. Rows indicate predictor variables: |VIS|, absolute velocity of isotherm shift for centroid shifts; GD, Genetic diversity; POS, location within the species range; Methods, the combined effect of all methodological variables included in the model (LogNTP, LogSAE, GS, DTy, SDes). Interactions are indicated by colon (":") between variable names. Columns indicate: Unique, the independent contribution of each term; Average share, the shared average contribution; Individual, combined contribution of unique and shared variance; Ind. perc. (%), percentage of total explained variance. The total variance explained by a single variable includes its unique contribution plus all shared contributions through interactions. For example, the variance explained by GD is calculated as 1.64 + 4.33 + 1.70 + 2.28 = 9.95% (with interaction explaining: 4.33 + 1.70 + 2.28 = 8.31%). The variance explained by |VIS| and POS are equal to 9.27% (interaction: 6.77%) and 38.04% (interaction: 9.5%), respectively.

	Unique	Average share	Individual	Ind. perc. (%)
|VIS|	0	0.0117	0.0117	2.4
GD	0	0.008	0.008	1.64
POS	0.1074	0.0318	0.1392	28.54
|VIS|:POS	-0.0066	0.0207	0.0141	2.89
GD:POS	0.0029	0.0182	0.0211	4.33
|VIS|:GD	0	0.0083	0.0083	1.7
|VIS|:GD:POS	-0.0009	0.012	0.0111	2.28
Methods	0.2402	0.0341	0.2743	56.23

Table S2. Summary of the effect estimates computed from the 10000 bootstrapped models. The model explains 50% of the species range shifts on average (95% CI from 47 to 53%). The * indicates significant values (P<0.05). Right-aligned variables indicate deviations from the left-aligned effect displayed above. Although multiple measurements of range shift may be available for the same species at different study areas, genetic diversity was treated as a species-level covariate. Refer to the methods session for further details on the modeling approach and included covariates.

Predictor variable	Avg. estimate [95% CI]	P-value
Intercept for centroid shifts with Abundance sampling of Occurrence-based data	-3.814 [-4.344;-3.342]	<0.0001*
Deviation for leading edge shifts	1.243 [1.128;1.354]	<0.0001*
Deviation for trailing edge shifts	0.572 [0.392;0.732]	<0.0001*
Deviation for abundance-based data	0.915 [0.78;1.056]	<0.0001*
Deviation for raw sampling	0.302 [0.209;0.394]	<0.0001*
Deviation for resurveyed sampling	-0.502 [-0.709;-0.313]	<0.0001*
Genetic diversity for centroid shifts	0.146 [0.084;0.204]	<0.0001*
Deviation for leading edge shifts	-0.163 [-0.244;-0.076]	0.0004*
Deviation for trailing edge shifts	-0.173 [-0.441;0.092]	0.0958
Velocity of isotherm shift for centroid shifts	0.308 [0.26;0.352]	<0.0001*
Deviation for leading edge shifts	-0.406 [-0.532;-0.289]	<0.0001*
Deviation for trailing edge shifts	0.085 [-0.148;0.299]	0.2085
Genetic diversity x Velocity of isotherm shift for centroid shifts	-0.086 [-0.122;-0.047]	0.0001*
Deviation for leading edge shifts	-0.042 [-0.124;0.05]	0.1626
Deviation for trailing edge shifts	-0.149 [-0.6;0.049]	0.123
Spatial resolution	0.065 [-0.005;0.126]	0.0336*

log(Spatial extent)	0.531 [0.455;0.617]	<0.0001*
log(Number of temporal units)	-0.067 [-0.122;-0.015]	0.004*

Table S3. Ranges of climate change velocities for which genetic diversity has a significant negative or positive effect on the velocity of species range shifts.

	Genetic diversity effect	
	Negative	Positive
Leading edge	2 to 5.8 km/yr	0 to 0.7 km/yr
Centroid	4.4 to 6.9 km/yr	0.1 to 3 km/yr
Trailing edge	2.7 to 5.6 km/yr	

Table S4. Summary of models testing whether latitude covaries with the relationship between range shift velocity, climate change velocity, genetic diversity, and range position. The baseline model includes all two- and three-way interactions among climate change velocity, genetic diversity, and range position. Additional models (Latitude 1 and Latitude 2) evaluate the role of latitude either as an additive covariate or through a full four-way interaction. Model performance is compared using R^2 and AIC values, with lower AIC indicating better fit and higher R^2 indicating greater explanatory power.

Model	$R^2_{Marginal}$	AIC
(Baseline)<br>Range shift velocity ~ |VIS| * GD * POS + Methods + (1 | Class)	48.8%	43.41
(Latitude 1)<br>Range shift velocity ~ |VIS| * GD * POS + Latitude + Methods + (1 | Class)	48.7%	45.41
(Latitude 2)<br>Range shift velocity ~ |VIS| * GD * POS * Latitude + Methods + (1 | Class)	37.9%	67.40

Table S5. Summary of models testing whether latitude, climate change velocity, and genetic diversity independently explain residual variation in species' range shift velocity. We first tested whether latitude explains the residuals of a baseline model that included climate change velocity, genetic diversity, range position, methodological variables (Methods), and a random effect of taxonomic class (Class) (that is, whether latitude accounts for variance not explained by these predictors). Next, we fitted a model including latitude and methodological variables, and a random effect of taxonomic class (Class), then tested whether climate change velocity and genetic diversity explain the residuals of this model (that is, the variance unexplained by latitude). R^2 values are reported for all models to indicate the proportion of variance explained. Overall, climate change velocity and genetic diversity explained more of the variation not captured by latitude than latitude explained of the variation not captured by climate change velocity and genetic diversity.

Model	$R^2_{Marginal}$
Residuals baseline ~ Latitude + (1 | Class)	0.59%
Residuals Latitude ~ Climate change velocity * Genetic diversity * Range position + (1 | Class)	6.48%

Dataset S1. Summary of the number of species and range shifts per taxonomic class, order and family.

Class	Order	Family	Species	Range shift
Insecta	Lepidoptera	Geometridae	176	262
Insecta	Lepidoptera	Noctuidae	158	280
Insecta	Lepidoptera	Drepanidae	12	21
Insecta	Lepidoptera	Zygaenidae	4	5
Insecta	Lepidoptera	Nymphalidae	36	110
Insecta	Lepidoptera	Sphingidae	11	15
Insecta	Lepidoptera	Pieridae	9	20
Insecta	Lepidoptera	Limacodidae	1	1
Insecta	Lepidoptera	Lycaenidae	12	61
Insecta	Lepidoptera	Hesperiidae	11	35
Insecta	Lepidoptera	Nolidae	8	15
Insecta	Lepidoptera	Erebidae	41	75
Insecta	Lepidoptera	Notodontidae	17	25
Insecta	Lepidoptera	Cossidae	3	4
Insecta	Lepidoptera	Lasiocampidae	5	7
Insecta	Lepidoptera	Riodinidae	1	1
Insecta	Lepidoptera	Papilionidae	5	8
Insecta	Lepidoptera	Pterophoridae	1	1

Insecta	Lepidoptera	Sesiidae	2	2
Insecta	Orthoptera	Gryllidae	1	1
Insecta	Orthoptera	Tettigoniidae	4	7
Insecta	Orthoptera	Acrididae	3	6
Insecta	Orthoptera	Tetrigidae	1	3
Insecta	Coleoptera	Dytiscidae	22	22
Insecta	Coleoptera	Carabidae	68	79
Insecta	Coleoptera	Coccinellidae	14	20
Insecta	Coleoptera	Hydrophilidae	5	5
Insecta	Coleoptera	Cerambycidae	2	2
Insecta	Coleoptera	Gyrinidae	1	1
Insecta	Coleoptera	Haliplidae	2	2
Insecta	Coleoptera	Helophoridae	5	5
Insecta	Coleoptera	Hydraenidae	1	1
Insecta	Coleoptera	Noteridae	1	1
Insecta	Coleoptera	Dryophthoridae	1	1
Insecta	Hemiptera	Pentatomidae	5	5
Insecta	Hemiptera	Corixidae	4	4
Insecta	Hemiptera	Coreidae	2	2
Insecta	Hemiptera	Rhopalidae	1	1

Insecta	Hemiptera	Gerridae	1	1
Insecta	Hemiptera	Hydrometridae	1	1
Insecta	Hemiptera	Veliidae	1	1
Insecta	Hemiptera	Notonectidae	3	3
Insecta	Hemiptera	Pleidae	1	1
Insecta	Hemiptera	Nepidae	1	1
Insecta	Odonata	Aeshnidae	7	21
Insecta	Odonata	Calopterygidae	3	9
Insecta	Odonata	Coenagrionidae	11	31
Insecta	Odonata	Cordulegastridae	1	3
Insecta	Odonata	Corduliidae	5	10
Insecta	Odonata	Libellulidae	14	35
Insecta	Odonata	Gomphidae	2	4
Insecta	Odonata	Lestidae	3	6
Insecta	Odonata	Platycnemididae	1	3
Insecta	Hymenoptera	Vespidae	1	1
Insecta	Hymenoptera	Andrenidae	31	53
Insecta	Hymenoptera	Megachilidae	22	29
Insecta	Hymenoptera	Apidae	68	206
Insecta	Hymenoptera	Pompilidae	3	3

Insecta	Hymenoptera	Crabronidae	28	28
Insecta	Hymenoptera	Colletidae	11	13
Insecta	Hymenoptera	Melittidae	4	9
Insecta	Hymenoptera	Formicidae	9	14
Insecta	Hymenoptera	Halictidae	35	51
Insecta	Hymenoptera	Mutillidae	1	1
Insecta	Diptera	Syrphidae	55	125
Insecta	Diptera	Stratiomyidae	5	5
Insecta	Diptera	Limoniidae	8	8
Insecta	Diptera	Tabanidae	2	2
Insecta	Diptera	Tipulidae	2	2
Insecta	Diptera	Pediciidae	1	1
Insecta	Blattodea	Ectobiidae	1	1
Insecta	Plecoptera	Leuctridae	2	2
Insecta	Plecoptera	Nemouridae	2	2
Aves	Passeriformes	Fringillidae	15	129
Aves	Passeriformes	Sturnidae	2	8
Aves	Passeriformes	Acrocephalidae	5	23
Aves	Passeriformes	Aegithalidae	2	7
Aves	Passeriformes	Icteridae	7	26

Aves	Passeriformes	Alaudidae	2	16
Aves	Passeriformes	Emberizidae	20	96
Aves	Passeriformes	Motacillidae	5	29
Aves	Passeriformes	Corvidae	12	66
Aves	Passeriformes	Artamidae	1	1
Aves	Passeriformes	Bombycillidae	1	3
Aves	Passeriformes	Calcariidae	3	19
Aves	Passeriformes	Troglodytidae	7	44
Aves	Passeriformes	Parulidae	25	100
Aves	Passeriformes	Cardinalidae	3	12
Aves	Passeriformes	Turdidae	11	72
Aves	Passeriformes	Certhiidae	2	22
Aves	Passeriformes	Cinclidae	2	3
Aves	Passeriformes	Nectariniidae	1	1
Aves	Passeriformes	Climacteridae	2	2
Aves	Passeriformes	Paridae	10	52
Aves	Passeriformes	Hirundinidae	4	23
Aves	Passeriformes	Mimidae	3	18
Aves	Passeriformes	Tyrannidae	6	28
Aves	Passeriformes	Petroicidae	2	2

Aves	Passeriformes	Muscicapidae	9	55
Aves	Passeriformes	Campephagidae	1	1
Aves	Passeriformes	Laniidae	3	12
Aves	Passeriformes	Locustellidae	1	3
Aves	Passeriformes	Maluridae	1	1
Aves	Passeriformes	Meliphagidae	1	1
Aves	Passeriformes	Oriolidae	1	4
Aves	Passeriformes	Panuridae	1	2
Aves	Passeriformes	Pardalotidae	1	1
Aves	Passeriformes	Passeridae	2	22
Aves	Passeriformes	Phylloscopidae	5	29
Aves	Passeriformes	Polioptilidae	1	9
Aves	Passeriformes	Prunellidae	1	10
Aves	Passeriformes	Regulidae	4	22
Aves	Passeriformes	Sittidae	3	16
Aves	Passeriformes	Sylviidae	5	34
Aves	Passeriformes	Vireonidae	5	16
Aves	Passeriformes	Zosteropidae	1	1
Aves	Accipitriformes	Accipitridae	9	55
Aves	Accipitriformes	Cathartidae	1	7

Aves	Accipitriformes	Pandionidae	1	9
Aves	Charadriiformes	Scolopacidae	21	102
Aves	Charadriiformes	Alcidae	5	8
Aves	Charadriiformes	Charadriidae	6	20
Aves	Charadriiformes	Laridae	9	27
Aves	Charadriiformes	Haematopodidae	1	3
Aves	Charadriiformes	Recurvirostridae	1	2
Aves	Charadriiformes	Stercorariidae	2	6
Aves	Strigiformes	Strigidae	9	38
Aves	Strigiformes	Tytonidae	1	6
Aves	Anseriformes	Anatidae	24	80
Aves	Coraciiformes	Alcedinidae	2	4
Aves	Galliformes	Phasianidae	9	43
Aves	Gruiformes	Rallidae	5	16
Aves	Gruiformes	Aramidae	1	1
Aves	Apodiformes	Trochilidae	1	4
Aves	Pelecaniformes	Ardeidae	5	17
Aves	Pelecaniformes	Threskiornithidae	1	3
Aves	Psittaciformes	Psittacidae	1	1
Aves	Piciformes	Picidae	11	61

Aves	Columbiformes	Columbidae	5	23
Aves	Cuculiformes	Cuculidae	2	5
Aves	Falconiformes	Falconidae	5	23
Aves	Suliformes	Sulidae	1	1
Aves	Caprimulgiformes	Caprimulgidae	1	2
Aves	Podicipediformes	Podicipedidae	3	9
Magnoliopsida	Sapindales	Sapindaceae	1	4
Magnoliopsida	Asterales	Asteraceae	16	73
Magnoliopsida	Asterales	Campanulaceae	3	9
Magnoliopsida	Asterales	Menyanthaceae	1	1
Magnoliopsida	Dipsacales	Adoxaceae	3	10
Magnoliopsida	Dipsacales	Caprifoliaceae	4	12
Magnoliopsida	Apiales	Apiaceae	5	18
Magnoliopsida	Apiales	Araliaceae	2	5
Magnoliopsida	Lamiales	Lamiaceae	11	33
Magnoliopsida	Lamiales	Plantaginaceae	13	61
Magnoliopsida	Lamiales	Orobanchaceae	2	4
Magnoliopsida	Lamiales	Oleaceae	1	1
Magnoliopsida	Lamiales	Phrymaceae	1	1
Magnoliopsida	Lamiales	Lentibulariaceae	1	3

Magnoliopsida	Lamiales	Scrophulariaceae	1	1
Magnoliopsida	Brassicales	Brassicaceae	7	19
Magnoliopsida	Ericales	Ericaceae	6	7
Magnoliopsida	Ericales	Primulaceae	3	15
Magnoliopsida	Caryophyllales	Caryophyllaceae	12	50
Magnoliopsida	Caryophyllales	Amaranthaceae	4	15
Magnoliopsida	Caryophyllales	Montiaceae	1	1
Magnoliopsida	Caryophyllales	Polygonaceae	10	37
Magnoliopsida	Fagales	Betulaceae	5	20
Magnoliopsida	Fagales	Fagaceae	1	3
Magnoliopsida	Fagales	Myricaceae	1	4
Magnoliopsida	Ranunculales	Ranunculaceae	6	23
Magnoliopsida	Ranunculales	Berberidaceae	2	2
Magnoliopsida	Ranunculales	Papaveraceae	1	1
Magnoliopsida	Solanales	Convolvulaceae	2	7
Magnoliopsida	Solanales	Solanaceae	1	5
Magnoliopsida	Saxifragales	Saxifragaceae	2	4
Magnoliopsida	Saxifragales	Grossulariaceae	2	5
Magnoliopsida	Myrtales	Onagraceae	4	22
Magnoliopsida	Myrtales	Lythraceae	2	4

Magnoliopsida	Rosales	Rosaceae	14	39
Magnoliopsida	Rosales	Rhamnaceae	1	3
Magnoliopsida	Rosales	Ulmaceae	1	1
Magnoliopsida	Rosales	Urticaceae	1	6
Magnoliopsida	Fabales	Fabaceae	15	54
Magnoliopsida	Malpighiales	Euphorbiaceae	1	1
Magnoliopsida	Malpighiales	Hypericaceae	2	7
Magnoliopsida	Malpighiales	Salicaceae	4	9
Magnoliopsida	Malpighiales	Violaceae	4	15
Magnoliopsida	Gentianales	Rubiaceae	5	19
Magnoliopsida	Gentianales	Gentianaceae	1	6
Magnoliopsida	Geraniales	Geraniaceae	4	16
Magnoliopsida	Boraginales	Hydrophyllaceae	1	1
Magnoliopsida	Malvales	Malvaceae	1	6
Magnoliopsida	Oxalidales	Oxalidaceae	1	3
Magnoliopsida	Vitales	Vitaceae	1	1
Polypodiopsida	Polypodiales	Pteridaceae	1	1
Polypodiopsida	Polypodiales	Dryopteridaceae	1	5
Polypodiopsida	Polypodiales	Cystopteridaceae	1	1
Polypodiopsida	Polypodiales	Thelypteridaceae	1	2

Polypodiopsida	Ophioglossales	Ophioglossaceae	1	1
Polypodiopsida	Equisetales	Equisetaceae	4	12
Polypodiopsida	Osmundales	Osmundaceae	1	1
Arachnida	Araneae	Araneidae	10	14
Arachnida	Araneae	Agelenidae	3	4
Arachnida	Araneae	Liocranidae	1	2
Arachnida	Araneae	Linyphiidae	68	112
Arachnida	Araneae	Lycosidae	11	17
Arachnida	Araneae	Amaurobiidae	2	3
Arachnida	Araneae	Theridiidae	10	16
Arachnida	Araneae	Hahniidae	1	2
Arachnida	Araneae	Cheiracanthiidae	1	1
Arachnida	Araneae	Clubionidae	6	11
Arachnida	Araneae	Cybaeidae	1	1
Arachnida	Araneae	Dictynidae	3	5
Arachnida	Araneae	Dysderidae	2	2
Arachnida	Araneae	Salticidae	6	12
Arachnida	Araneae	Gnaphosidae	4	6
Arachnida	Araneae	Tetragnathidae	7	13
Arachnida	Araneae	Thomisidae	3	4

Arachnida	Araneae	Philodromidae	7	11
Arachnida	Araneae	Phrurolithidae	1	2
Arachnida	Araneae	Pisauridae	1	2
Arachnida	Araneae	Segestriidae	1	2
Arachnida	Araneae	Miturgidae	1	2
Arachnida	Opiliones	Phalangiidae	4	4
Liliopsida	Poales	Poaceae	24	84
Liliopsida	Poales	Cyperaceae	11	24
Liliopsida	Poales	Juncaceae	2	6
Liliopsida	Poales	Typhaceae	2	5
Liliopsida	Asparagales	Orchidaceae	2	2
Liliopsida	Asparagales	Iridaceae	1	5
Liliopsida	Asparagales	Asparagaceae	3	3
Liliopsida	Alismatales	Araceae	2	2
Liliopsida	Alismatales	Potamogetonaceae	1	2
Liliopsida	Alismatales	Juncaginaceae	2	8
Liliopsida	Liliales	Melanthiaceae	1	1
Liliopsida	Liliales	Colchicaceae	1	1
Actinopterygii	Clupeiformes	Clupeidae	8	14
Actinopterygii	Clupeiformes	Engraulidae	2	2

Actinopterygii	Perciformes	Ammodytidae	1	1
Actinopterygii	Perciformes	Anarhichadidae	2	4
Actinopterygii	Perciformes	Callionymidae	1	1
Actinopterygii	Perciformes	Carangidae	2	2
Actinopterygii	Perciformes	Serranidae	1	2
Actinopterygii	Perciformes	Chiasmodontidae	1	1
Actinopterygii	Perciformes	Pomacentridae	1	1
Actinopterygii	Perciformes	Sciaenidae	3	5
Actinopterygii	Perciformes	Sparidae	3	5
Actinopterygii	Perciformes	Trachinidae	1	1
Actinopterygii	Perciformes	Zoarcidae	3	6
Actinopterygii	Perciformes	Latridae	1	1
Actinopterygii	Perciformes	Stichaeidae	2	3
Actinopterygii	Perciformes	Odacidae	1	1
Actinopterygii	Perciformes	Haemulidae	1	1
Actinopterygii	Perciformes	Stromateidae	1	2
Actinopterygii	Perciformes	Labridae	2	2
Actinopterygii	Perciformes	Pomatomidae	1	2
Actinopterygii	Perciformes	Scombridae	2	2
Actinopterygii	Perciformes	Tetragonuridae	1	1

Actinopterygii	Perciformes	Trichiuridae	1	2
Actinopterygii	Perciformes	Trichodontidae	1	1
Actinopterygii	Perciformes	Mullidae	1	1
Actinopterygii	Anguilliformes	Anguillidae	1	2
Actinopterygii	Anguilliformes	Synaphobranchidae	1	1
Actinopterygii	Gadiformes	Moridae	1	1
Actinopterygii	Gadiformes	Gadidae	9	23
Actinopterygii	Gadiformes	Macrouridae	4	4
Actinopterygii	Gadiformes	Lotidae	2	3
Actinopterygii	Gadiformes	Merlucciidae	2	9
Actinopterygii	Gadiformes	Phycidae	3	17
Actinopterygii	Stomiiformes	Stomiidae	4	4
Actinopterygii	Pleuronectiformes	Bothidae	1	1
Actinopterygii	Pleuronectiformes	Paralichthyidae	4	8
Actinopterygii	Pleuronectiformes	Pleuronectidae	16	35
Actinopterygii	Pleuronectiformes	Scophthalmidae	1	2
Actinopterygii	Pleuronectiformes	Soleidae	1	1
Actinopterygii	Scorpaeniformes	Triglidae	2	2
Actinopterygii	Scorpaeniformes	Cyclopteridae	1	1
Actinopterygii	Scorpaeniformes	Psychrolutidae	2	3

Actinopterygii	Scorpaeniformes	Cottidae	2	6
Actinopterygii	Scorpaeniformes	Sebastidae	7	13
Actinopterygii	Scorpaeniformes	Hemitripteridae	1	2
Actinopterygii	Scorpaeniformes	Agonidae	2	4
Actinopterygii	Scorpaeniformes	Hexagrammidae	3	3
Actinopterygii	Tetraodontiformes	Diodontidae	1	1
Actinopterygii	Tetraodontiformes	Tetraodontidae	1	1
Actinopterygii	Tetraodontiformes	Monacanthidae	1	1
Actinopterygii	Myctophiformes	Myctophidae	4	4
Actinopterygii	Beryciformes	Trachichthyidae	1	1
Actinopterygii	Osmeriformes	Bathylagidae	1	1
Actinopterygii	Osmeriformes	Osmeridae	2	7
Actinopterygii	Lophiiformes	Lophiidae	2	5
Actinopterygii	Stephanoberyciformes	Melamphaidae	2	2
Actinopterygii	Salmoniformes	Salmonidae	1	2
Actinopterygii	Batrachoidiformes	Batrachoididae	1	1
Actinopterygii	Syngnathiformes	Syngnathidae	1	1
Actinopterygii	Zeiformes	Zeidae	2	2
